# Structural Characterization of the Interaction of Human Lactoferrin with Calmodulin

**DOI:** 10.1371/journal.pone.0051026

**Published:** 2012-12-06

**Authors:** Jessica L. Gifford, Hiroaki Ishida, Hans J. Vogel

**Affiliations:** Biochemistry Research Group, Department of Biological Sciences, University of Calgary, Calgary, Alberta, Canada; University of Oldenburg, Germany

## Abstract

Lactoferrin (Lf) is an 80 kDa, iron (Fe^3+^)-binding immunoregulatory glycoprotein secreted into most exocrine fluids, found in high concentrations in colostrum and milk, and released from neutrophil secondary granules at sites of infection and inflammation. In a number of cell types, Lf is internalized through receptor-mediated endocytosis and targeted to the nucleus where it has been demonstrated to act as a transcriptional *trans*-activator. Here we characterize human Lf’s interaction with calmodulin (CaM), a ubiquitous, 17 kDa regulatory calcium (Ca^2+^)-binding protein localized in the cytoplasm and nucleus of activated cells. Due to the size of this intermolecular complex (∼100 kDa), TROSY-based NMR techniques were employed to structurally characterize Ca^2+^-CaM when bound to intact apo-Lf. Both CaM’s backbone amides and the ε-methyl group of key methionine residues were used as probes in chemical shift perturbation and cross-saturation experiments to define the binding interface of apo-Lf on Ca^2+^-CaM. Unlike the collapsed conformation through which Ca^2+^-CaM binds the CaM-binding domains of its classical targets, Ca^2+^-CaM assumes an extended structure when bound to apo-Lf. Apo-Lf appears to interact predominantly with the C-terminal lobe of Ca^2+^-CaM, enabling the N-terminal lobe to potentially bind another target. Our use of intact apo-Lf has made possible the identification of a secondary interaction interface, removed from CaM’s primary binding domain. Secondary interfaces play a key role in the target’s response to CaM binding, highlighting the importance of studying intact complexes. This solution-based approach can be applied to study other regulatory calcium-binding EF-hand proteins in intact intermolecular complexes.

## Introduction

**Figure 1:**
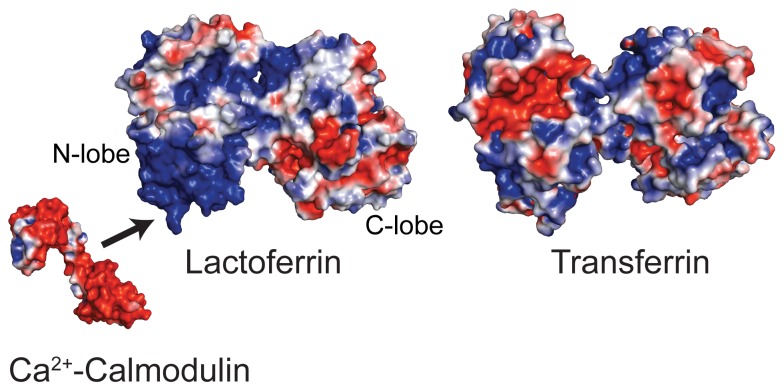
Surface structure of Ca^2+^-CaM, Fe^3+^-Lf, and Fe^3+^-Tf with the electrostatic surface potential on each indicated. Negatively charged CaM (*red*) would be attracted to the positively charged N-terminus of Lf (*blue*). Unlike Lf, Tf lacks the extremely positively-charged N-terminus. PDB codes 1CLL, 1B0L, and 1H76, respectively.

In humans, lactoferrin (Lf) is a cell-secreted, 80 kDa host defense glycoprotein with antibacterial, antiviral, antifungal, antitumor, and anti-inflammatory activities [1], [2]. Like other members of the transferrin (Tf) protein family, Lf binds a single ferric iron cation (Fe^3+^) to each of its two homologous domains, the N- and C-domains, with extremely high affinity (*K_a_* ∼10^20^ M^−1^) (Figure 1) [3]. Lf is secreted in its Fe^3+^-free (apo) form by epithelial cells into most exocrine fluids, or alternatively, is released at a very high concentration from the secondary granules of activated neutrophils recruited to sites of inflammation. Several of Lf’s biological activities are associated with this protein’s ability to sequester Fe^3+^, starving invading pathogens of an essential nutrient as well as preventing the formation of harmful host-generated reactive oxygen species [4]. Fe^3+^-independent antimicrobial activities have also been ascribed to Lf. These activities are linked to the ability of this highly cationic (pI ∼ 9) protein, in particular its N-terminal lactoferricin domain, to interact with negatively charged molecular and cellular components of both host cells and pathogens [5].

Lf has also been shown to indirectly and directly modulate the activity of innate and adaptive immune cells [6], [7], [8], [9]. By binding to, and thus neutralizing pro-inflammatory bacterial molecules, Lf prevents the release of cytokines responsible for both the activation and the recruitment of leukocytes in inflamed tissues [10], [11]. Furthermore, Lf’s affinity for glycosaminoglycans prevents leukocyte activation by blocking the chemokine-proteoglycan interaction required for signal transduction through G-protein coupled receptors [12], [13]. Lf has also been shown to directly promote the maturation and differentiation of dendritic cells [14], and T- and B-lymphocytes [15], [16]. The presence of specific Lf receptors on immune cells (as well as other cell types) and their demonstrated capability to bind to and internalize Lf through clatherin-mediated endocytosis suggests that Lf can act intracellularly [8], [17]. A number of experiments demonstrate that once inside the cell, these receptors target Lf to the nucleus where it is thought to act as a transcriptional activator or transcription factor, modulating the activity of immune and epithelial cells at the genetic level [15], [18], [19], [20], [21], [22], [23].

The interaction between Lf and the intracellular calcium (Ca^2+^)-binding protein calmodulin (CaM) was first reported by de Lillo et al [24]. Expressed in all cell types and localized to the cytoplasm and nucleus, CaM is a small, acidic, bilobal protein in which the N- and C-terminal lobes are connected by a highly flexible linker (Figure 1) [25]. Each lobe of CaM contains a pair of helix-loop-helix EF-hands [26], and upon binding Ca^2+^ through these motifs undergoes a conformation change that enables this protein to bind to and regulate numerous cell signaling effectors involved in growth, proliferation and movement [27], [28]. The interaction between CaM and Lf was shown to be Ca^2+^-dependent and Fe^3+^-independent, unique in the Tf family to Lf, and likely to involve Lf’s N-terminal lactoferricin domain. In this study, we have explored this interaction further, characterizing the thermodynamic properties of this interaction as well as the structural factors defining Ca^2+^-CaM in an ∼100 kDa complex with apo-Lf. While numerous studies of CaM with target peptides derived from CaM-binding domains (CBDs) exist, currently there is only limited structural information available for complexes of Ca^2+^-CaM and intact target proteins [29], [30], [31]. In order to obtain structural information about Lf-bound CaM we have used a combined NMR spectroscopy approach [32], [33]. Ours represents the largest CaM complex studied to date by solution NMR spectroscopy, and our method can easily be adapted to study Ca^2+^-CaM bound to other intact target proteins.

## Materials and Methods

### Sample Preparation

CaM was overexpressed and purified from Escherichia coli BL21(DE3) cells containing the pET30b(+) expression vector. Besides unlabelled CaM used for the biophysical characterization of the interaction, this study required four isotopically enriched versions of CaM for the structural work. The backbone chemical shift assignments of free or Lf-bound CaM were completed using either uniformly ^13^C/^15^N- or ^2^H/^13^C/^15^N-labeled CaM prepared in M9 media containing 0.5 g/L ^15^NH_4_Cl, 4 g/L [^13^C_6_]glucose, in H_2_O or 99.9% ^2^H_2_O, respectively. A methionine methyl-labeled version of otherwise fully deuterated CaM, (^1^H/^13^C-methyl-Met)/^2^H/^15^N CaM, was created by supplementing 100 mg/L ^1^H-α,ε-^13^C-ε-^2^H-methionine to 99.9% ^2^H_2_O M9 media containing 0.5 g/L ^15^NH_4_Cl and 4 g/L [^2^H_7_]glucose one hour prior to induction with IPTG [34], [35]. Finally, uniformly ^2^H/^15^N-labeled CaM was prepared in 99.9% ^2^H_2_O M9 media with 0.5 g/L ^15^NH_4_Cl and 4 g/L ^2^H_6_-glucose. The E139Q and E139R mutants of CaM were generated using the QuickChange site-directed mutagenesis kit (Stratagene) and confirmed by DNA sequencing. All versions of CaM were purified to homogeneity by Ca^2+^-dependent phenyl-Sepharose column chromatography as previous described [36], [37]. Unlabelled and ^2^H/^15^N-labelled CaM C-terminal lobe (residues 78–148) was produced through controlled tryptic digestion of either unlabelled or ^2^H/^15^N-labelled Ca^2+^-CaM and purified according to previously established protocols [38], [39], with the exception that the two lobes of CaM were separated on a Sephacryl S200 (GE Healthcare) gel filtration column instead of by hydrophobic interaction chromatography. The identity of the C-terminal lobe was confirmed by UV absorbance spectroscopy monitoring CaM’s two Tyr amino acid residues found solely in this lobe (Tyr-99 and -138). The concentrations of CaM and TR2C were determined using ε_276_ = 2900 M^−1^cm^−1^ and ε_276_ = 3006 M^−1^cm^−1^, respectively [40].

CaM and its mutants were dansylated using dansyl chloride (Sigma) and a previously established protocol [41]. Excess dansyl chloride was removed by a desalting Sephadex G-25 column (Amersham Biosciences) equilibrated with 20 mM NH_4_HCO_3_ followed by lyophilization. The CaM concentration after dansylation was measured using the Bio-Rad protein assay (Bio-Rad Lab.). Absorption measurements using ε_320_ = 3400 M^−1^cm^−1^ showed an incorporation of ∼1.2 mol dansyl chloride/mol CaM.

Human Lf was recombinantly expressed in *Aspergillus awamori* and was made available as a kind gift from Agennix [42]. The protein was provided as a lyophilized powder and demonstrated to contain a homogeneous sample of apo-Lf through differential scanning calorimetry and SDS PAGE. Fe^3+^-Lf was prepared using an “iron saturation” protocol [43]. Briefly, apo-Lf was dissolved in 50 mM Tris (pH 7.5), 3 mM FeCl_3_, 3 mM nitrilotriacetic acid, 3 mM NaHCO_3_, and 100 mM KCl to a concentration of 10 mg/mL. The mixture was incubated at room temperature for 18 h, desalted on a Sephadex G-25 column equilibrated with 20 mM NH_4_HCO_3_, and lyophilized. The concentration of Lf was determined using the extinction coefficients, ε_280_ = 85 700 M^−1^cm^−1^ for apo-LF, and ε^1%^_465_ = 0.58 for Fe^3+^-Lf. Tf was obtained from Sigma-Aldrich.

### Gel Mobility Shift Assay

Non-denaturing polyacrylamide gel mobility shift electrophoresis was performed following a previously established protocol [44], except that the running buffer and samples contained 0.1 and 0.5 mM CaCl_2_, respectively, and, due to the denaturation of both apo- and Fe^3+^-Lf in the presence of 4 M urea [45], the urea was excluded.

### Fluorescence Spectroscopy

All fluorescence spectra were recorded on a Varian Cary Eclipse spectrofluorimeter. In each experiment the dansyl group attached to wild-type or mutant versions of CaM was selectively excited at 340 nm and emission spectra were recorded from 400 to 550 nm. All samples contained 1.4 µM dansylated wild-type or mutant E139Q or E139R CaM in 50 mM Tris (pH 7.5), 1 mM CaCl_2_, and either 50, 100, or 150 mM KCl. Titration experiments involved sequential addition of microliter volumes of ∼80 µM Lf in the respective buffer into 1 ml samples of wild-type or mutant CaM. As Fe^3+^-Lf absorbs light at both the excitation and emission wavelengths of the dansyl group, the emission spectra were corrected for the inner filter effect using the equation:
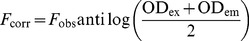
(1)and extinction coefficients of ε_340_ = 1.95×10^4^ M^−1^cm^−1^ and ε_485_ = 4.15×10^3^ M^−1^cm^−1^, for the excitation and emission wavelengths, respectively [46]. The changes in fluorescence intensity at 485 nm for each complex were used to calculate the dissociation constant (*K*_d_) for the interaction. Curve fitting was performed using a one site model in the CaLigator software [47]. Each titration was performed in triplicate.

### NMR Spectroscopy

NMR samples contained 0.5–1 mM ^13^C/^15^N−, ^2^H/^13^C/^15^N−, (^1^H/^13^C-methyl-Met)/^2^H/^15^N-, or ^2^H/^15^N-labeled CaM alone or mixed with a 1.25∶1 molar excess of unlabelled apo-Lf in 20 mM Bis-Tris (pH 6.8), 150 mM KCl, 4 mM CaCl_2_, 0.03% NaN_3_, 0.5 mM 2,2-dimethyl-2-silapentaine-5-sulfonate (DSS) and either 90% H_2_O/10% ^2^H_2_O, 20% H_2_O/80% ^2^H_2_O, or 99.9% ^2^H_2_O. All NMR experiments were performed at 37°C on a Bruker Avance 500 or 700 MHz spectrometer equipped with a triple resonance inverse cryogenic probe. The backbone chemical shift assignments of Ca^2+^-CaM in the absence of Lf were obtained using the standard 2D [^15^N, ^1^H]-HSQC and 3D CBCA(CO)NH, HNCACB, HN(CA)CO, and HNCO experiments. The backbone chemical shift assignments of Ca^2+^-CaM bound to apo-Lf were obtained using a [^15^N, ^1^H]-TROSY-HSQC, [^15^N, ^1^H]-TROSY-HNCA, [^1^H, ^1^H]-NOESY-[^15^N, ^1^H]-TROSY-HSQC, and [^15^N, ^1^H]-TROSY-HNCO. A mixing time of 300 ms was used for the [^1^H, ^1^H]-NOESY-[^15^N, ^1^H]-TROSY-HSQC experiment. Methyl group chemical shift perturbations (CSPs) of CaM’s methionine amino residues upon the binding of apo-Lf were observed in [^13^C, ^1^H]-HMQC spectra using CaM selectively protonated on the methionine methyl group [(^1^H/^13^C-methyl-Met)/^2^H/^15^N-labelled CaM].

Two different cross-saturation transfer experiments were performed. Cross saturation of CaM’s backbone amides when complexed with unlabelled apo-Lf was monitored using ^2^H/^15^N-labeled CaM and a TROSY cross-saturation transfer experiment [33]. Selective irradiation of the aliphatic region of the ^1^H NMR spectrum was accomplished through an adiabatic WURST-2 pulse with a total sweep width of 3.6 ppm centered at 0.9 ppm. The saturation was highly selective for aliphatic protons and no noticeable saturation of the H_2_O resonance was observed. A high concentration of ^2^H_2_O (80%) was used to avoid excessive spin diffusion that can occur in α-helical proteins such as CaM. Spectra were recorded with saturation times (T_sat_) of 0 and 1.0 s. In the second experiment, apo-Lf-induced cross-saturation of the ε-methyl group of CaM’s nine methionines was determined using (^1^H/^13^C-methyl-Met)/^2^H/^15^N-labelled CaM and a methyl-utilizing cross-saturation experiment [48], [49]. Selective irradiation was achieved through a Reburp pulse. Due to residual protonation of the sample (the Hα position of the methyl-labeled methionine employed remains protonated [34]), the irradiation was centered at 0.0 ppm with a sweep width of 2.0 ppm. T_sat_ of 0 and 3.0 s were used with an interscan delay of 2 s. For both experiments, a ratio comparing intensity with and without saturation was calculated for each resonance.

Chemical shifts in all spectra were referenced using DSS to obtain ^1^H, ^15^N and ^13^C chemical shifts as previously described [50]. All spectra were processed using NMRPipe [51] and were analyzed using the NMR-View software [52]. The ^13^Cα values obtained from ^2^H/^13^C/^15^N-labelled CaM were increased by 0.5 ppm to compensate for isotope shifts caused by the directly attached deuterium atom [53]. CSPs in the [^15^N-^1^H]-TROSY-HSQC spectrum of Ca^2+^-CaM complexed with apo-Lf were calculated as the weighted average chemical shift difference of the ^1^H and ^15^N resonances according to the equation [54]:
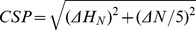
(2)


## Results

### Ca^2+^-CaM Binds Stoichiometrically to Apo- or Fe^3+^-Lf with a Micromolar Affinity

**Figure 2:**
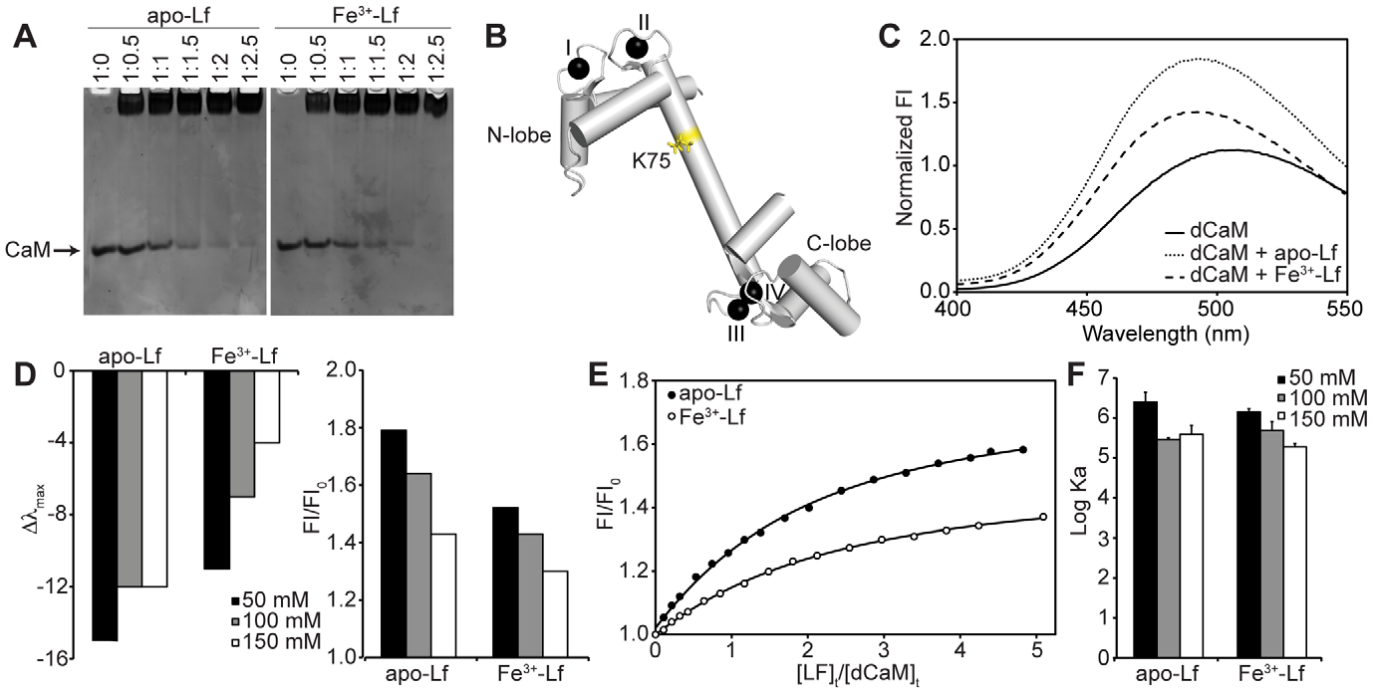
Biophysical Characterization of the Interaction Between Ca^2+^-CaM and Lf. **A)** Non-denaturing PAGE band shift analysis for Ca^2+^-CaM binding to apo- and Fe^3+^-Lf. The ratio of CaM to Lf is indicated above each lane. **B)** Ribbon representation of Ca^2+^-CaM with the side chain of Lys-75 highlighted (PDB code 1CLL). The dansyl fluorophore is attached to the side chain of this residue. **C)** Fluorescence emission spectra of Ca^2+^-dCaM in the absence and presence of apo- or Fe^3+^-Lf in a solution of 100 mM KCl. **D)** Effect of increasing KCl concentration on the wavelength maximum (*left*) and intensity (*right*) of the fluorescence emission of dCaM when bound to apo- or Fe^3+^-Lf. **E)** Titration of dCaM with apo- or Fe^3+^-Lf as monitored through dansyl fluorescence in a solution of 100 mM KCl. The relative fluorescence intensities are plotted against the ratio of the total concentration of Lf and dCaM. **F)** Log *K_a_* values displayed as bars for the binding of dCaM to apo- or Fe^3+^-Lf in solutions of varying KCl concentration. Values are represented as mean ± SEM for three independent titration experiments.

Gel band shift assays were used to initially assess the interaction between Ca^2+^-CaM and apo- or Fe^3+^-Lf. This technique is typically used to examine low molecular weight peptide-CaM complexes [55], [56], [57], [58], but here was applied to the ∼100 kDa CaM:Lf interaction. Increasing amounts of apo- or Fe^3+^-Lf were incubated with a constant amount of Ca^2+^-CaM and subsequently analyzed. The addition of either apo- or Fe^3+^-Lf corresponded with the appearance of a high molecular weight band and the disappearance of the free Ca^2+^-CaM band (Figure 2A). In assays with apo- or Fe^3+^-Lf, the CaM band has mostly disappeared upon the addition of 1.5 molar equivalents of Lf, suggesting that Ca^2+^-CaM forms a 1∶1 complex with both states of Lf.

The interaction between apo- or Fe^3+^-Lf and Ca^2+^-CaM was next studied through fluorescence spectroscopy by measuring changes in the fluorescence emission spectrum of dansylated CaM (dCaM). Covalently bound to Lys-75 in CaM’s central linker, the dansyl fluorophore is a sensitive probe to study the interaction between CaM and its binding partners (Figure 2B) [41], [59]. The addition of apo- or Fe^3+^-Lf to Ca^2+^-saturated dCaM caused a significant increase in dansyl fluorescence intensity as well as a shift in the emission wavelength maximum of several nm to shorter wavelengths (blue shift), both characteristic of the dansyl entering a more hydrophobic environment (Figures 2C and D). Interestingly, both of these effects were greater upon the addition of apo-Lf to Ca^2+^-dCaM, as compared to Fe^3+^-Lf, suggesting a link between the Fe^3+^-bound state of Lf and the binding environment of Ca^2+^-dCaM. In contrast, the addition of the related protein Tf did not have any effect on the emission spectrum of Ca^2+^-dCaM (results not shown). Titrations were performed to establish the binding affinity between apo- or Fe^3+^-Lf and Ca^2+^-dCaM. The resulting curves are hyperbolic and produced *K_d_* values of 3.5 and 2.1 µM for interactions with apo- and Fe^3+^-Lf at an ionic strength of 0.1 M, respectively (Figure 2E and F). These curves fit well to a one-site model agreeing with the 1∶1 stoichiometry suggested by the band shift assay.

To test the role of ionic interactions in the binding of apo- or Fe^3+^-Lf to Ca^2+^-dCaM, the fluorescence titrations were performed at varying salt concentrations (Figures 2D and F). Independent of the Fe^3+^-state of Lf, both the fluorescence emission intensity as well as the blue shift in the emission wavelength maximum showed a negative dependence on ionic strength (Figure 2D). For apo and Fe^3+^-Lf, this change in the fluorescence emission profile of dCaM corresponded to a decrease in binding affinity for Ca^2+^-dCaM (Figure 2F), pointing to the involvement of ionic in addition to hydrophobic interactions in the binding of both forms of Lf to Ca^2+^-dCaM.

### Structural Properties of Ca^2+^-CaM in the Apo-Lf:Ca^2+^-CaM Complex

An NMR-based approach was used to structurally characterize Ca^2+^-CaM when bound to Lf as well as determine the interaction interface. Lactoferrin-bound Fe^3+^ is paramagnetic, causing significant line-width broadening and thus signal loss in NMR spectra (data not shown). Because of this, we have focused our structural studies on the Ca^2+^-CaM:apo-Lf complex. Our initial titration of ^15^N-labeled CaM with apo-Lf produced spectra with the line-width broadening characteristic of a large intermolecular interaction with an unfavorable correlation time (not unexpected since the complex has a molecular weight of ∼100 kDa). To sharpen the line-widths of CaM when bound to Lf, uniformly ^2^H/^13^C/^15^N-labeled CaM and TROSY-based NMR experiments were employed. The titration of ^2^H, ^15^N-labeled Ca^2+^-CaM with unlabeled apo-Lf produced TROSY-HSQC spectra describing an intermolecular interaction occurring in slow chemical exchange: the ^1^H(^15^N) backbone resonances corresponding to free Ca^2+^-CaM disappear as those for the protein in complex with apo-Lf appear (data not shown). As slow exchange is typically observed for intermolecular interactions with a dissociation constant on the order of 10^−6^ M or less, this type of chemical exchange agrees with the Ca^2+^-CaM:apo-Lf affinity obtained through fluorescence spectroscopy. Furthermore, only a single set of CaM resonances was observed when bound to apo-Lf, pointing to a 1∶1 stoichiometry [60].

To investigate the structural basis of CaM’s interaction with Lf, we assigned the backbone chemical shifts of free Ca^2+^-CaM and Ca^2+^-CaM when bound to apo-Lf. For free Ca^2+^-CaM, the chemical shift assignments were obtained using standard double- and triple-resonance NMR experiments conducted on uniformly ^15^N- and ^13^C/^15^N -labeled protein samples. ^1^H, ^13^Cα, ^13^C′, and ^15^N resonance assignments of Ca^2+^-CaM in complex with apo-Lf were obtained from 2D [^15^N-^1^H]-TROSY-HSQC, and 3D TROSY-HNCA, TROSY-HNCO, and [^1^H-^1^H]-NOESY-[^15^N-^1^H]-TROSY-HSQC spectra. The backbone chemical shifts of CaM were determined with 95 and 85% completion for free Ca^2+^-CaM and Ca^2+^-CaM in complex with apo-Lf, respectively.

The secondary structure of Ca^2+^-CaM when bound to apo-Lf was assessed by comparing the ^13^Cα and ^13^C′ chemical shifts obtained from the spectra to a database of random coil values (Figure S1, top). For comparison, ^13^Cα and ^13^C′ chemical shifts were used to determine the secondary structures of free Ca^2+^-CaM or Ca^2+^-CaM complexed with a peptide representing the CBD of CaM-dependent kinase I (CaMKI) (Figure S1, middle and bottom) [34]. In all three instances, Ca^2+^-CaM adopts the same secondary structural elements: two domains of a pair of helix-loop-helix motifs connected by a short linker.

### Identification of the Apo-Lf Binding Surface on Ca^2+^-CaM

**Figure 3:**
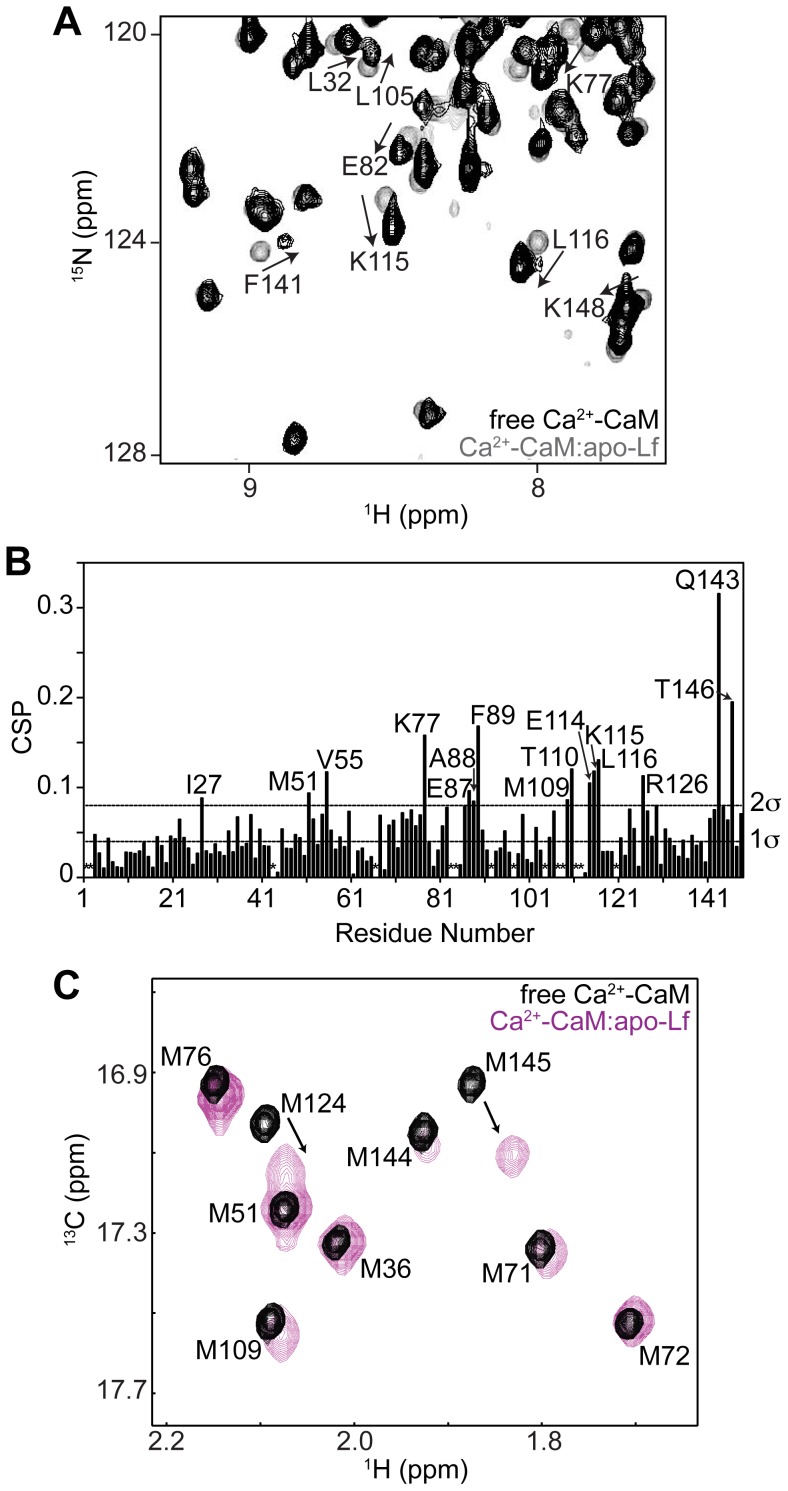
Binding of Ca^2+^-CaM to Apo-Lf Enacts Changes in Chemical Shifts of the C-lobe of CaM. **A)** CSPs in TROSY-HSQC spectra of Ca^2+^-CaM upon binding apo-Lf. A selected region of the spectra are shown. **B)** CSPs between bound and unbound ^2^H/^15^N -labeled Ca^2+^-CaM plotted as a function of amino acid residue. The two horizontal lines on the graph represent standard deviations in chemical shift changes. (*) Residues with missing assignments. **C)** [^13^C-^1^H]-HMQC spectrum of Ca^2+^-bound (^1^H/^13^C-methyl-Met)/^2^H/^15^N CaM either free or bound to apo-Lf. Due to the selective labeling scheme, only the ε-methyl groups of CaM’s methionines are detected. Eight of CaM’s nine methionine amino acid residues line the hydrophobic binding pockets of Ca^2+^-CaM. Significant changes in chemical shift are seen for the ε-methyls of methionine groups found in CaM’s C-terminus of CaM.

Structural changes in Ca^2+^-CaM induced upon binding apo-Lf were probed by comparing the backbone ^1^H and ^15^N chemical shifts of free Ca^2+^-CaM and Ca^2+^-CaM bound to apo-Lf. Although the majority of CaM’s ^1^H(^15^N) cross peaks in a TROSY-HSQC spectrum did not change upon the addition of unlabelled apo-Lf, several signals are clearly shifted (Figure 3A). Differences in these chemical shifts were quantified and the CSPs plotted as function of residue number (Figure 3B). Although a few backbone resonances in the N-lobe (residues 1–76) and the central linker of CaM (residues 77–79) were affected by the binding of apo-Lf, most changes map to CaM’s C-lobe (residues 80–148).

In most structurally characterized Ca^2+^-CaM complexes, CaM interacts with the primarily hydrophobic CBD of the target protein through extensive hydrophobic patches exposed in each lobe as a result of Ca^2+^ binding. More than any other type of amino acid in these patches, the methionine side chains, in particular their terminal ε-methyl groups, are directly involved in target binding [34], [35], [55], [61], [62], and their presence has been shown through mutant-based studies to be essential [63], [64]. Using CaM ^13^C, ^1^H-labeled on the methionine ε-methyl group but otherwise isotopically ^2^H and ^12^C, [(^1^H/^13^C-methyl-Met)/^2^H/^15^N CaM], the high number of methionine residues in CaM (9 out of 148 amino acid residues) as well as their location in the hydrophobic pockets was exploited to serve as probes for defining the binding interface of apo-LF on Ca^2+^-CaM [34], [35]. Agreeing with the ^1^H(^15^N) CSPs observed in the TROSY-HSQC spectrum of apo-Lf-bound Ca^2+^-CaM, the methyl groups of methionines found in the N-terminal lobe of CaM (Met-36 and Met-51) experienced little change in chemical shift upon the addition of apo-Lf (Figure 3C). In contrast, methyl groups of methionines found in CaM’s C-terminal lobe, in particular Met-124 and Met-145, experienced a significant change in ^13^C and ^1^H chemical shifts.

**Figure 4:**
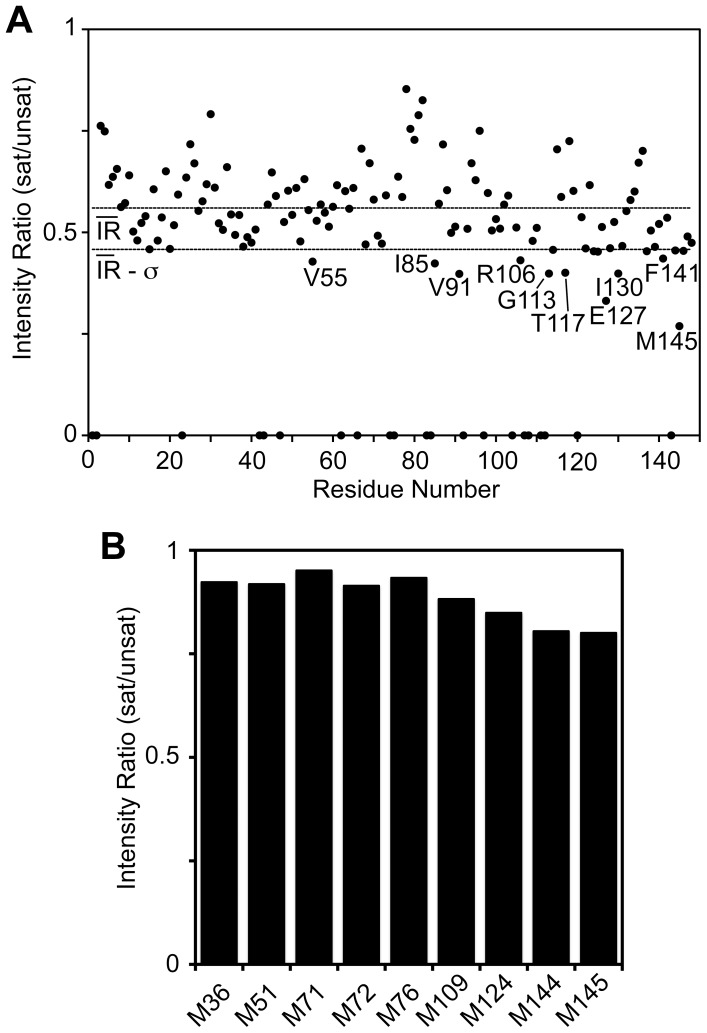
Identification of Apo-Lf Contacts on Ca^2+^-CaM from Cross-Saturation Data. **A)** Plot of residue-specific cross-saturation-induced amide proton signal intensity changes for apo-Lf-bound Ca^2+^-CaM. The unassigned residues are shown as having an intensity ratio of zero. The two horizontal lines on the graph represent the statistical significance of the change seen for an individual residue. **B)** Cross-saturation-induced methionine ε-methyl signal intensity changes for apo-Lf-bound Ca^2+^-CaM.

NMR cross-saturation experiments were next employed to further define the binding interface of apo-Lf on Ca^2+^-CaM [33]. In the first experiment, ^2^H/^15^N-labeled CaM was mixed with saturating amounts of unlabeled apo-Lf. By applying radio-frequency pulses to the methyl/methylene region of the proton spectrum, sites in the unlabeled Lf were saturated, and this saturation transferred across to the nearest ^1^H(^15^N) pairs in labeled CaM by spin diffusion. The ratios of TROSY-HSQC cross-peak intensities, with and without saturation, provide a measure of the proximity of various ^1^H(^15^N) pairs to the interface. Cross-saturation experiments were recorded with saturation periods of 1.0 s before the experiment started. Figure 4A shows a plot of TROSY-HSQC saturated/unsaturated signal intensity ratios for Ca^2+^-CaM in complex with apo-Lf. Because of peak overlap in the spectrum, only 95% of assigned cross-peaks could be analyzed. Although all ^1^H(^15^N) cross-peaks in the TROSY-HSQC spectrum experienced a decrease in signal intensity, strongly cross-saturated residues (intensity ratio <0.46) were found predominantly in the C-lobe of Ca^2+^-CaM. In the second experiment, apo-Lf-induced cross-saturation of the ε-methyl group of CaM’s nine methionine residues was determined using (^1^H/^13^C-methyl-Met)/^2^H/^15^N-labeled CaM. Again all ^1^H(^13^C) cross-peaks in the [^13^C, ^1^H]-HMQC spectrum experienced a decrease in signal intensity, however the intensity loss was greater for methionine residues found in the C-terminal lobe of CaM (Figure 4B).

### Structural Comparison Suggests Ca^2+^-CaM Adopts a Non-canonical Conformation when Bound to Apo-Lf

**Figure 5:**
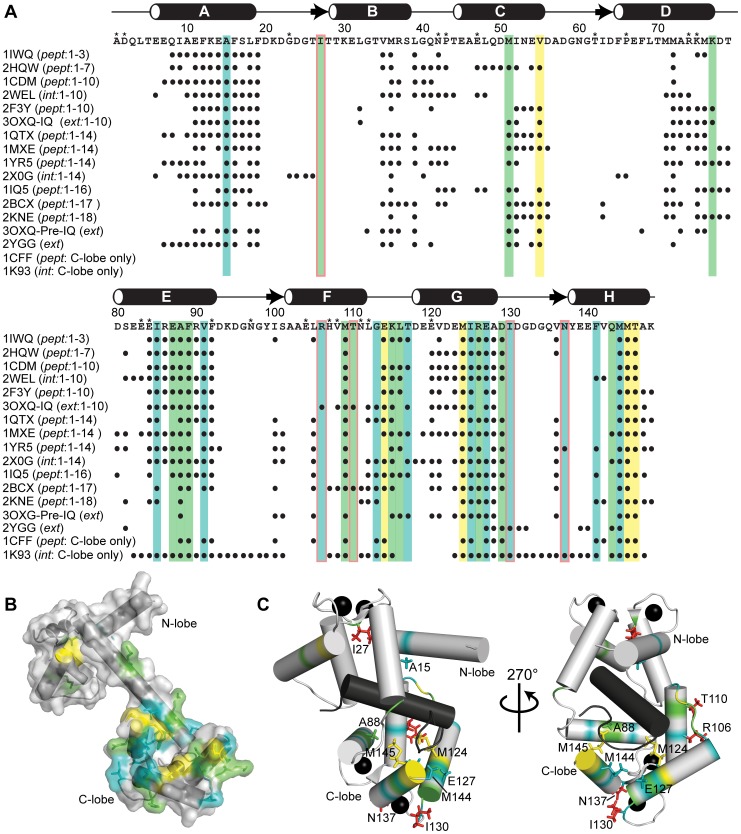
Comparison of the Ca^2+^-CaM:Apo-Lf Interface with Other Known Ca^2+^-CaM:Target Interfaces. **A)** Summary of the CSP and saturation transfer data depicted on the primary sequence of CaM. Residues perturbed upon binding of apo-Lf are highlighted in *green*, cross-saturated are shown in *blue*, those that experience both CSP and cross-saturation in *yellow*, and missing residues by an *. *Black circles* indicate CaM residues for which amide atoms are found within 6.5 Å from the target peptide or protein in the indicated CaM complex deposited in the PDB. A *red box* highlights residues involved in the interaction with apo-Lf but uncommonly involved in interaction with other targets. The structures as indicated by their PDB codes are as follows: 1IWQ: MARCKs, 2HQW: NMDA receptor, 1CDM: CaMKIIα, 2WEL: CaMKIIδ, 2F3Y: CaV1.2 Ca^2+^ channel IQ domain motif, 3OXQ: CaV1.2 Ca^2+^ channel pre-IQ/IQ domain, 1QTX: smMLCK, 1MXE: CaMKI, 1YR5 and 2X0G: DAPK, 1IQ5: CaMKK, 2BCX: ryanodine receptor, 2KNE: plasma membrane Ca^2+^ pump, 2YGG: Na^+^/H^+^ exchanger NHE1, 1CFF: plasma membrane Ca^2+^ pump, 1K93: *B. anthracis* oedema factor. For each, whether or not the CBD is represented by a peptide (*pept*), extended sequence (*ext*), or the intact protein (*int*) is indicated. The classification of the CaM-binding motif is also shown. The secondary structure of Ca^2+^-CaM is indicated above: cylinders and arrows indicate α-helix and β-strand, respectively. CaM amino acid residues experiencing a significant change in chemical shift or cross-saturation upon binding apo-Lf are mapped on the structure of **B)** free Ca^2+^-CaM (PDB code 1CLL) or **C)** Ca^2+^-CaM bound to the CBD of CaMKK (PDB code: 1IQ5). In both, the same coloring scheme as in A is employed. Highlighted side chains reflect amino acid residues commonly found at the interaction interface, except those colored in *red* which reflect residues rarely involved in the binding interface.

The outcome of the CSP and cross-saturation transfer experiments are summarized in Figures 5A and B in which residues experiencing a normalized CSP>0.8 and cross-saturation with an intensity ratio of <0.46 are depicted together with the amino acid sequence of CaM or on the structure of free Ca^2+^-CaM, respectively. From this analysis it is clear that the majority of residues affected upon interaction with apo-Lf are located in the C-lobe of CaM: helices E-H and the loops between helices F and G, and G and H. In contrast, the majority of residues in the N-terminal lobe remain unperturbed suggesting the N-terminal lobe does not play a key role in the binding interface. This observation that the C-terminal lobe of CaM is sufficient for the interaction with apo-Lf is supported by gel band shift analysis as well as TROSY-HSQC titration data of the CaM:apo-Lf interaction using the proteolytically isolated C-terminal lobe of CaM (Figure S2).

To further characterize the interaction of apo-Lf with Ca^2+^-CaM, CaM complex structures deposited in the Protein Data Bank (PDB) were analyzed for CaM residues with amide atoms found within 6.5 Å from the target peptide or protein. The rational being, atoms within 6.5 Å would likely display CSP, cross-saturation, or both upon complex formation. As revealed by this global comparison, many of the CaM residues affected upon apo-Lf binding are contact residues in the structures of other CaM complexes. Apo-Lf binds to the C-lobe of CaM in a manner not unlike that seen in other complexes, predominantly through the methionine-rich, hydrophobic surface exposed as a result of Ca^2+^-binding. In contrast, unlike most CaM complex structures apo-Lf does not engage the typically observed contact regions of CaM’s N-terminal lobe, particularly the A- and D-helices.

Of the CaM-target complexes deposited in the PDB, the interaction between Ca^2+^-CaM and apo-Lf shares a significant number of contacts, particularly in CaM’s C-terminal lobe, with those observed in the crystal structure of Ca^2+^-CaM bound to a peptide representing the CBD of CaM-dependent kinase kinase (CaMKK) [65]. CaM residues that experience significant cross-saturation or CSP upon binding apo-Lf have been highlighted on this structure (Figure 5C). From this mapping, the involvement of the hydrophobic surface of the C-terminal lobe of CaM in the interaction is clear, as well as the positions of residues that are not typically found at the binding interface of Ca^2+^-CaM complexes. Of the complexes surveyed, Ile-27 in the N-terminal lobe and C-terminal lobe residues Arg-106, Thr-110, Ile-130, and Asn-137 were rarely, if ever found to be within 6.5 Å of the target peptide or protein. These residues are positioned on the outside of the CaM molecule, away from the hydrophobic pockets that form the primary interface.

### Characterization of Apo-Lf’s Secondary Interface on Ca^2+^-CaM

**Figure 6:**
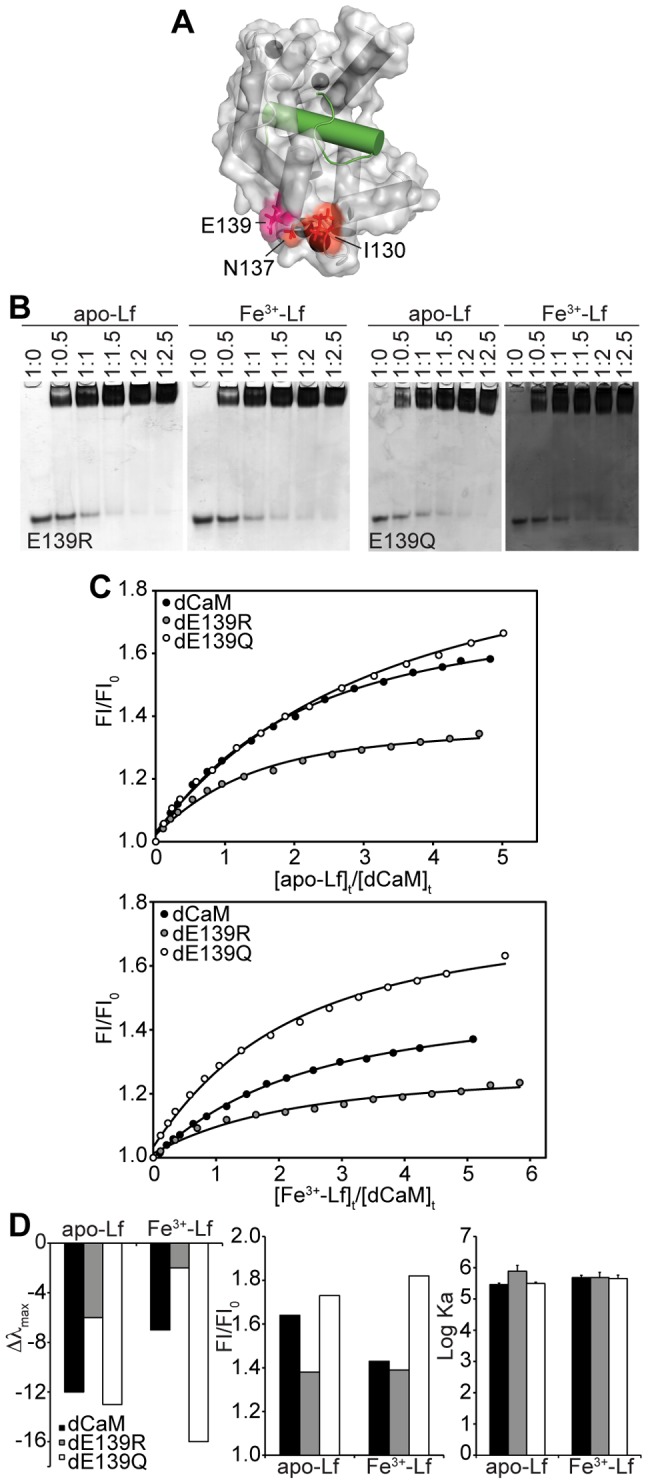
Disrupting the Secondary Interface. **A)** Residues that make up a secondary interface (*red*) as identified from CSP and cross-saturation data are indicated on the structure of Ca^2+^-CaM (*grey,* surface representation) bound to the CaMKK peptide (*green, helix*). The position of the mutant studied, Glu-139 is indicated (*deep pink*). **B)** Non-denaturing PAGE band shift analysis of the interaction of CaM mutants E139R and E139Q with apo/Fe^3+^-Lf. The ratio of CaM to Lf is indicated above each lane. **C)** Steady-state fluorescence titration of wild-type and mutant dCaMs with apo- or Fe^3+^-Lf. The relative fluorescence intensities are plotted against the ratio of total Lf and dCaM concentrations. **D)** Fluorescence parameters and derived binding constants for apo- or Fe^3+^-Lf binding to wild-type and mutant dCaMs in 100 mM KCl. Error bars represent SEM for three independent titrations.

Amino acid residues Arg-106 and Thr-110 of CaM’s C-terminal lobe are found near Met-109 in the F-helix, a known interaction CaM:target interface. In contrast, Ile-130 and Asn-137 are located on the outside of the CaM’s C-terminal lobe, an area typically not involved in CaM:peptide complexes (Figures 5A and 6A). This interface makes up much of the fourth Ca^2+^-binding EF-loop (the loop and small β-sheet between helices G and H), and as such, residues in this loop have to be carefully mutated [26]. To avoid affecting the Ca^2+^-binding ability of the fourth EF-hand, Glu-139 was chosen to investigate the role of this interface in CaM’s interaction with both apo- and Fe^3+^-Lf. Glu-139 was replaced with the charge neutral Gln (E139Q) or the positively charged Arg (E139R), and the impact of the mutagenesis assessed by gel band shift assay and fluorescence titration.

Band shift assays indicate that CaM mutants E139Q and E139R bind to apo- and Fe^3+^-Lf in a stoichiometric manner (Figure 6B), similar to the 1∶1 ratios seen with the wild-type protein (Figure 2A). When studied through fluorescence spectroscopy, the exchange of a glutamate amino acid residue for a glutamine in dE139Q did not have much affect on the binding ability and affinity of apo-Lf for Ca^2+^-CaM as a similar shift in wavelength maximum and increase in fluorescence intensity were observed for dE139Q and dCaM, and a comparable intermolecular affinity was calculated for the interaction (Figures 6C and D). In contrast, the binding of Fe^3+^-Lf to dE139Q resulted in a significant increase in observed maximum wavelength shift and fluorescence intensity when compared to the wild-type dCaM. However, despite affecting the fluorescence characteristics of the interaction, the replacement of this glutamate with a glutamine had no significant effect on the affinity between these two proteins. The outcome with dE139R contrasts that of dE139Q. Compared to wild-type dCaM, the observed blue shift as well as fluorescence intensity enhancement of dE139R upon binding either apo- or Fe^3+^-Lf is reduced. Regardless of the different characteristics of the dansyl emission spectra, the glutamate to arginine substitution had no significant affect on the affinity of either apo or Fe^3+^-Lf for Ca^2+^-CaM as determined through fluorescence titration. This is consistent with the results of the band shift assay depicted in Figure 6B.

## Discussion

Previous studies have shown that CaM is capable of binding apo- and Fe^3+^-Lf in a Ca^2+^-dependent manner [24]. Here, we have examined at a biophysical and structural level the interaction between these two proteins. Gel band shift assays and fluorescence spectroscopy studies indicate that the protein-protein complex forms with a 1∶1 stoichiometry (Figures 2A and E), a binding ratio supported by TROSY-HSQC titrations (results not shown), and a comparable intermolecular affinity for both apo- and Fe^3+^-Lf. Despite these similarities, differences in the emission profile of a dansyl fluorophore attached to CaM suggest Ca^2+^-CaM interacts with the two forms of Lf through distinct mechanisms. The change in dansyl fluorescence intensity as well as wavelength maximum of dCaM observed upon binding either apo- or Fe^3+^-Lf is strongly influenced by salt concentration (Figure 2D): increasing ionic strength caused a decrease in the blue shift observed upon complex formation. The dansyl group of dCaM is attached predominantly to the side chain of Lys-75, located in the central linker of CaM (Figure 2B) [59], and the observed changes in the fluorescence intensity are most likely caused by differences in the local environment around Lys-75. Although a greater blue shift has been observed for dCaM when bound to Ca^2+^-CaM-binding proteins of other systems [66], our results with dCaM are consistent with those seen with the 5-hydroxytryptamine2A receptor [57] and a peptide representing the CBD of the cell cycle inhibitor p21 [58], suggesting a possible common binding mode with these systems that have yet to be fully structurally characterized.

Analysis of ^13^Cα and ^13^C′ chemical shifts of ^2^H/^13^C/^15^N-labelled Ca^2+^-CaM in complex with apo-Lf revealed little change in CaM secondary structure when compared to both free CaM or the protein bound to a peptide representing the CBD of CaMKI (Figure S1). This is consistent with previously determined structures of Ca^2+^-CaM complexes [61]. Ca^2+^-CaM forms intermolecular contacts through its amino acid side chains not the backbone of the protein, and as a result it is predominantly the orientation of CaM’s two domains that adjust between the different structures and not the overall fold of CaM itself. The central linker that connects the two domains is highly flexible in the absence of a target protein and facilitates this adjustment. In many cases, residual dipolar couplings (RDCs) can be used to orient the two domains of CaM with respect to each other [34], [35], [67], however, for the Ca^2+^-CaM:apo-Lf complex, the alignment media required to collect RDCs appeared to interact with Lf itself, preventing this type of analysis.

Perhaps reflecting the micromolar affinity of this interaction (Figure 2F), a comparison of CaM in the presence and absence of apo-Lf revealed CSPs for only a subset of signals (Figure 3A and B). More drastic spectral alterations are typically observed for Ca^2+^-CaM:target peptide complexes. The calculated average chemical shift difference between free Ca^2+^-CaM [68] and Ca^2+^-CaM bound to the CaMKI peptide [69] or the skeletal myosin light chain kinase (skMLCK) peptide [70] is 0.28 and 0.27 ppm, respectively. In contrast, this number is only 0.05 ppm for the Ca^2+^-CaM:apo-Lf interaction. These smaller observed CSPs may suggest CaM is sampling a large conformational heterogeneity even when bound to apo-Lf analogous to what has been shown in CaM complexes with other proteins or peptides [71], [72]. To map the binding interface of apo-Lf on Ca^2+^-CaM, we have used CSP (Figure 3) and saturation transfer experiments (Figure 4). These experiments have looked at both the changes affecting CaM’s backbone amides as well as the ε-methyl groups of key methionine residues. The residues affected by these experiments are highlighted on the primary sequence of CaM in Figure 5, and the regions identified by the CSP data are in good agreement with the regions identified through cross-saturation experiments. Both data sets along with gel band shift assays and TROSY-HSQC titrations performed using the C-terminal lobe CaM alone (Figure S2) point to the C-terminal lobe of CaM as the major interaction interface. Along with a number of hydrophobic and hydrophilic amino acid residues, this data also identifies a number of charged residues as being involved in the interaction (Lys-77, Glu-87, Arg-106, Glu-114, Lys-115, Arg-126, and Glu-127), accounting for this complex’s apparent sensitivity to an increased ionic strength.

**Figure 7:**
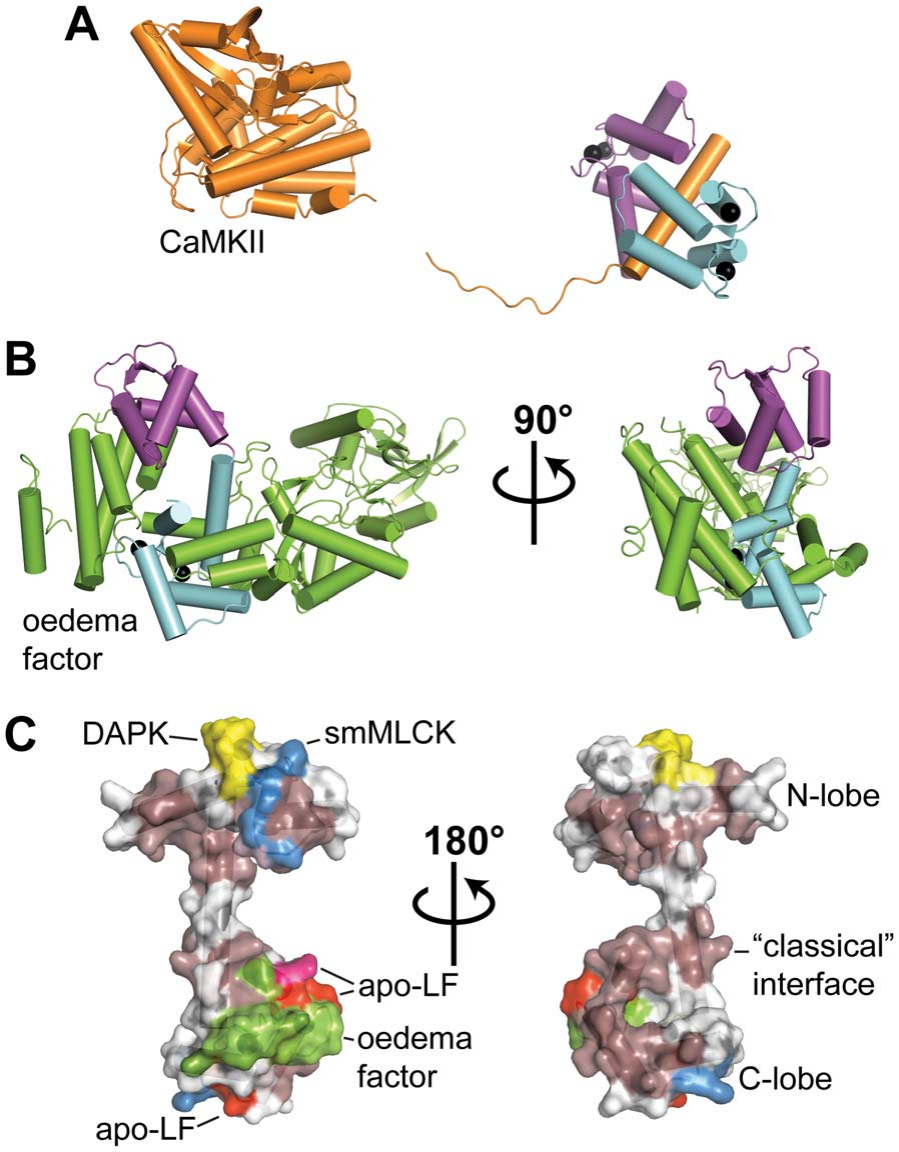
Comparison of the Secondary Interface of Apo-Lf to that of Other Complexes. From X-ray structures, ribbon representation of CaM bound to: **A)** intact CaMKIIδ (PDB code: 2WEL) or **B)** intact *B. anthracis* oedema factor (PDB code: 1K93). In each, the N- and C-lobes of CaM are colored *purple* and *light blue*, respectively. CaMKIIδ and oedema factor are colored *orange* and *green*, respectively. **C)** Secondary interfaces seen for apo-Lf or in other intact complexes (including DAPK, PDB code: 2X0G) are mapped onto the surface of free Ca^2+^-CaM and delineated by color. Glu-139 is highlighted in *deep pink*. The secondary interface of apo-Lf overlaps with that of the oedema factor.

Figure 5A also identifies the contact regions for a number Ca^2+^-CaM:target complexes deposited in the PDB. The contact regions of apo-Lf on the C-terminal lobe Ca^2+^-CaM are in agreement with those seen in other complexes; importantly, all critical methionine amino acid residues found in the C-terminal lobe of CaM demonstrated methyl cross-saturation, and either backbone CSP, backbone cross-saturation, or both. Contrasting with these results, few residues in CaM’s N-terminal lobe experienced a CSP>0.8 ppm upon binding apo-Lf (Ile-27, Met-51, and Val-55), and of these, only the backbone amide of Val-55 demonstrated cross-saturation. As seen in Figure 5A, the A-helix of CaM is typically located in close proximity to the target peptide/protein. The lack of significant engagement of the A-helix suggests CaM’s conformation when bound to apo-Lf is not the classical compact mode employed when binding many CaM-activated protein kinases (Figures 5C and 7A). Instead CaM appears to assume an extended conformation with the C-terminal lobe as the major binding interface and the N-terminal lobe interacting only superficially. In this respect, the Ca^2+^-CaM:apo-Lf complex displays structural similarities with that of CaM bound to the *Bacillus anthracis* oedema factor. In the X-ray structure of this complex, CaM adopts an elongated conformation with an α-helix of the oedema factor occupying the hydrophobic pocket of CaM’s C-terminal lobe, and the N-terminal lobe is not a major interaction interface (Figure 7B) [29]. However, unlike the CaM:oedema factor complex in which the N-terminal lobe of CaM does not bind Ca^2+^, when bound to apo-Lf the four signals corresponding to glycine residues 25, 61, 98, and 134 found in the four Ca^2+^-binding loops are observed at low field (>10 ppm), indicating that Ca^2+^ is bound in each EF-hand. In this regard, CaM’s interaction with apo-Lf belongs to a growing class of CaM complexes in which the protein:protein interaction is formed predominately through CaM’s C-terminal lobe [29], [73], [74], [75], [76], [77].

Our approach of studying CaM’s interaction with intact apo-Lf instead of a peptide representation of the CBD enables us to identify and study potential secondary interaction interfaces. To date, structural information on CaM bound to an intact target exists for only a handful of complexes, all but one studied through X-ray crystallography. While for some, the CBD is independent from the rest of the protein and peptide-representation is sufficient (CaMKI [69], CaMKIIδ [31]), for others additional contacts on CaM are found (oedema factor [29], DAPK [30]) and have been shown to play an essential role in target enzyme activation (MLCK [78], [79]) (Figure 7C). In addition to the hydrophobic face of CaM’s C-terminal lobe, apo-Lf forms contacts with an acidic region near Glu-139 on the outside of this lobe (Figure 5A and 6A). To examine this secondary interface we generated two CaM mutants, one removing the negative charge of this amino acid (E139Q), and one introducing a positive charge (E139R). As compared to the wild-type protein, the removal of a negative charge had no effect on the binding affinity of Ca^2+^-CaM for apo-Lf, and a limited effect on the fluorescence emission spectrum of the attached dansyl group (Figure 6D). In contrast, the introduction of a positive charge into the secondary interface greatly reduced the binding-induced blue shift and intensity increase of the dansyl group’s fluorescence emission spectrum, but had no effect on the calculated binding affinity between these two proteins. The introduction of a positive charge on an acidic face of CaM appears to disrupt or reorient apo-Lf’s secondary interaction interface, ultimately affecting the chemical environment of the dansyl group. However, because this point mutation does not affect CaM’s primary binding interface, that involving the hydrophobic patches of the C-terminal lobe, there is no substantial effect on the overall affinity of the interaction. Analogous to apo-Lf, X-ray structures indicate this same acidic face of CaM’s C-terminal lobe is an important contact area for both intact oedema factor and an extended sequence of the Na^+^/H^+^ exchanger NHE1 [29], [80] (Figures 5A and 7C).

Although we lack structural details of the interaction between Ca^2+^-CaM and Fe^3+^-Lf, our fluorescence spectroscopy experiments point to a Fe^3+^-dependence in Ca^2+^-CaM’s binding interface on Lf. The blue-shift and intensity increase in the emission spectrum of Ca^2+^-dCaM when bound to Fe^3+^-Lf is less than that observed when bound to apo-Lf suggesting the dansyl group is in a different environment (Figures 2C and D), and the significant effect of the E139Q mutation on the emission spectrum points to a larger role of this negatively charged group in the secondary interface (Figures 6C and D). These observed differences in the binding behavior of Ca^2+^-CaM for apo- and Fe^3+^-Lf could be explained by the 54° “open to closed” conformational transition experienced by each lobe of Lf upon binding Fe^3+^ [3]. Due to favorable surface characteristics, ability to block antimicrobial activity [24], and selectivity for Lf over Tf (Figure 1, results not shown), CaM likely binds the lactoferricin domain of Lf. This region of Lf, found in the N-terminal subdomain of Lf’s N-lobe, is known to bind a number of other proteins partners [81], [82], and an Fe^3+^-dependent change in conformation here could explain the observed differences in the fluorescence emission spectra.

Despite its initial classification as an extracellular, secreted protein, there is increasing evidence for an intracellular role for Lf. Circulating levels of Lf increase dramatically during the inflammatory process [83] and intact Lf has been detected in the fecal matter of infants [84], suggesting a significant portion survives digestion at least in the developing gut. Specific, saturable Lf receptors (LfRs) have been detected on the plasma membrane of several cell types including enterocytes, bronchial epithelial, brain, mammary gland, monocytes, macrophages, lymphocytes, mast, and cytotrophoblasts [85], [86], [87], [88], [89], [90], [91]. Upon binding apo- or Fe^3+^-Lf, the Lf:LfR complex is internalized through clathrin-mediated endocytosis and in a number of cell types, targeted to the nucleus through the N-terminal subdomain of the N-lobe [20], [21], [92], [93], [94], [95]. The nuclear localization of Lf suggests a role in transcriptional regulation and indeed, Lf has been shown to *trans*-activate AP-1 and NFκB, two transcription factors that play central roles in the inflammatory response, and control of cell division, differentiation and apoptosis [15], [18], [89], [93], [96]. Lf has also been found bound to DNA in the nucleus [97], where it can bind to specific DNA sequences [23], and it has been demonstrated to function as a transcription factor increasing the expression of *IL1B* [98] and *TGF-β1* [93]. These pro-inflammatory effects are contrasted with the anti-inflammatory activity exhibited by Lf’s ability to repress endothelial cell TNF-α-induced *ICAM-1* transcription by occupying the binding site for NF-κB in this gene’s promoter [92]. Further highlighting the role of Lf as a transcriptional activator, an alternative isoform, ΔLf, is expressed solely in the cell and serves as a transcription factor controlling the expression of several genes involved in the cell cycle and apoptosis [99], [100].

Similar to Lf, CaM plays a key role in the signal transduction cascades that lead to cell differentiation and proliferation [101]. CaM targeting to the nucleus is a Ca^2+^-dependent process requiring the increased cytoplasmic Ca^2+^ concentration that occurs in response to cell stimulation. Due to its size, Ca^2+^-CaM diffuses freely through nuclear pores and it has been proposed that nuclear CaM-binding proteins serve as a sink for this protein, leading to the accumulation of CaM in the nucleus in response to an elevation of intracellular free Ca^2+^ [102]. In the nucleus, CaM activates CaMKII and IV, the CaM-dependent phosphatase calcineurin, and relieves inhibition of the ubiquitous pro-proliferation transcription factor MEF2 by CaM-interacting corepressors Cabin1 and class II histone deacetylases. The Ca^2+^-dependence of the CaM:Lf interaction as reported by de Lillo et al., Lf’s nuclear targeting, and the role of Ca^2+^ in the internalization of Lf [86], point to the physiological significance of the macromolecular complex detailed here. The lack of Lf’s interaction with the N-terminal lobe of CaM suggests that CaM could act as an “adaptor protein”, binding Lf through interactions with its C-terminal lobe and another molecule with the N-terminal lobe. This mechanism of action is akin to that ascribed for CaM in complex with the *Nicotiana tabacum* mitogen-activated protein kinase phosphatase (NtMKP1) [77]. Like apo-Lf, NtMKP1 interacts weakly with the N-terminal lobe of CaM and this interaction would easily be replaced by that of another target protein of higher affinity. On its own, the isolated N-terminal lobe of CaM is capable of binding other CBD peptides [56], [103], [104], as well as intact target proteins [105]. Furthermore, related EF-hand proteins have been shown to act as adaptor proteins including centrin and Ca^2+^- and integrin-binding protein 1 [106]. Finally, Ca^2+^-CaM itself can facilitate the movement of large molecules into the nucleus [107], [108], potentially playing a key role in the nuclear targeting of Lf.

### Conclusions

Here, we have characterized at the molecular level the interaction between Lf and CaM, a protein:protein interaction at the intersection of the host-defense pathways and the Ca^2+^ signaling network. In the activated cell, Lf and CaM coexist in the nucleus at sufficient concentrations to form a complex. Using TROSY-based NMR techniques as well as strategic isotope labeling, we have identified the binding regions of Ca^2+^-CaM involved in forming an ∼100 kDa macromolecular complex with apo-Lf. At present, this is the largest CaM/target complex studied by solution NMR methods. By analyzing CaM’s interaction with intact Lf, instead of the more common technique of using peptide-representation of the CBD, we have identified a key secondary binding interface away from the hydrophobic pockets of each lobe. The roles of secondary binding interfaces continue to expand [29], [30], [78], [79], and our approach can be used to glean structural information on other CaM complexes with intact target proteins or large catalytically active fragments in solution, thereby more clearly elucidating CaM-target regulatory mechanisms. Furthermore, it can be applied to study the protein:target interactions of other regulatory EF-hand calcium-binding proteins.

## Supporting Information

Figure S1 **NMR Analysis of Ca^2+^-CaM Secondary Structure When Bound to Apo-Lf.** Secondary structure analysis based on ^13^Cα and ^13^C′ chemical shifts of Ca^2+^-CaM bound to either apo-Lf, CaMKIp, or in the absence of a target protein. The plotted function is the difference of the secondary shifts of both nuclei [Δδ(^13^Cα)+Δδ(^13^C′)], represented as bars. Positive chemical shift deviations from random coil values are characteristic of α-helices, while negative deviations described the extended structures of β-sheets and coil. *Closed circles* indicate residues excluded from analysis due to missing chemical shift assignments. Perceived difference in chemical shift in several residues in the C-lobe of CaM in the Ca^2+^-CaM:apo-Lf complex are due to missing assignments. Secondary structure elements identified in all three CaM structures are shown schematically at the top.
(TIF)

Figure S2 **On its own, the C-terminal domain of CaM is sufficient to interact apo-Lf. A)** Non-denaturing PAGE band shift analysis of the isolated C-terminal lobe Ca^2+^-CaM binding to apo-Lf. The ratio of CaM to apo-Lf is indicated above each lane. **B)** NMR titration data examining the binding of unlabeled apo-Lf to the isolated C-terminal lobe of ^2^H/^15^N-labeled Ca^2+^-CaM. TROSY-HSQC spectra were collected at titration steps corresponding to the [apo-Lf]/[CaM C-lobe] molar ratio of 0 (*black*), 0.5 (*red*), 0.75 (*yellow*), 1 (*blue*), 1.25 (*green*). These spectra are characteristic of an interaction in fast exchange, contrasting with the slow exchange observed in the titration of intact CaM. This difference in chemical exchange likely reflects the loss of the minor stabilizing contributions provided by the N-terminal lobe of CaM.
(TIF)
