# Supplementary figures and images for: Structural Characterization of the Interaction of Human Lactoferrin with Calmodulin
Source: PLoS One. 2012 Dec 6;7(12):e51026. doi: 10.1371/journal.pone.0051026 (PMC3516504; doi:10.1371/journal.pone.0051026)

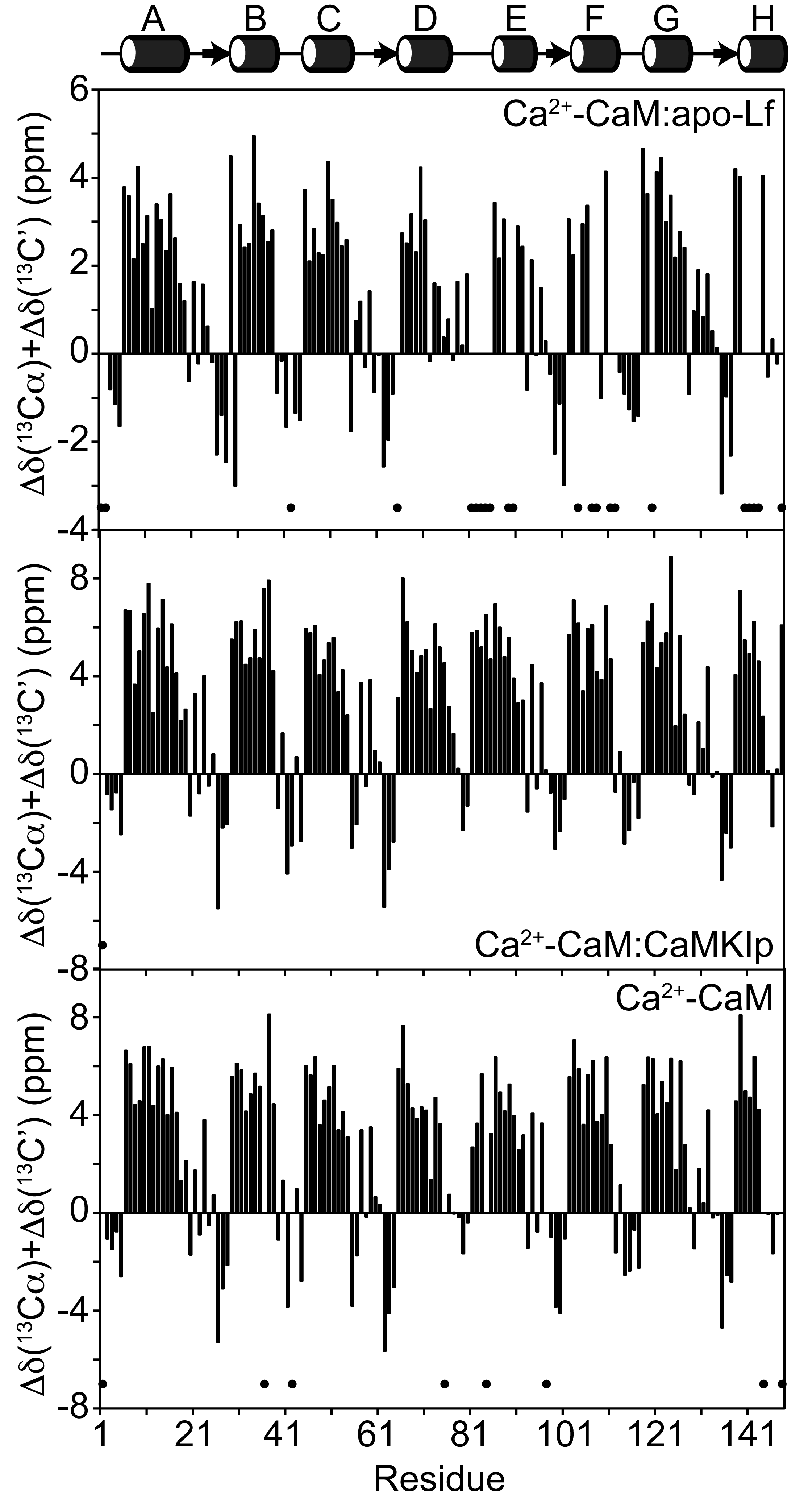

Supplement: Figure S1 — NMR Analysis of Ca2+-CaM Secondary Structure When Bound to Apo-Lf. Secondary structure analysis based on 13Cα and 13C′ chemical shifts of Ca2+-CaM bound to either apo-Lf, CaMKIp, or in the absence of a target protein. The plotted function is the difference of the secondary shifts of both nuclei [Δδ(13Cα)+Δδ(13C′)], represented as bars. Positive chemical shift deviations from random coil values are characteristic of α-helices, while negative deviations described the extended structures of β-sheets and coil. Closed circles indicate residues excluded from analysis due to missing chemical shift assignments. Perceived difference in chemical shift in several residues in the C-lobe of CaM in the Ca2+-CaM:apo-Lf complex are due to missing assignments. Secondary structure elements identified in all three CaM structures are shown schematically at the top. (TIF) [file pone.0051026.s001.tif]

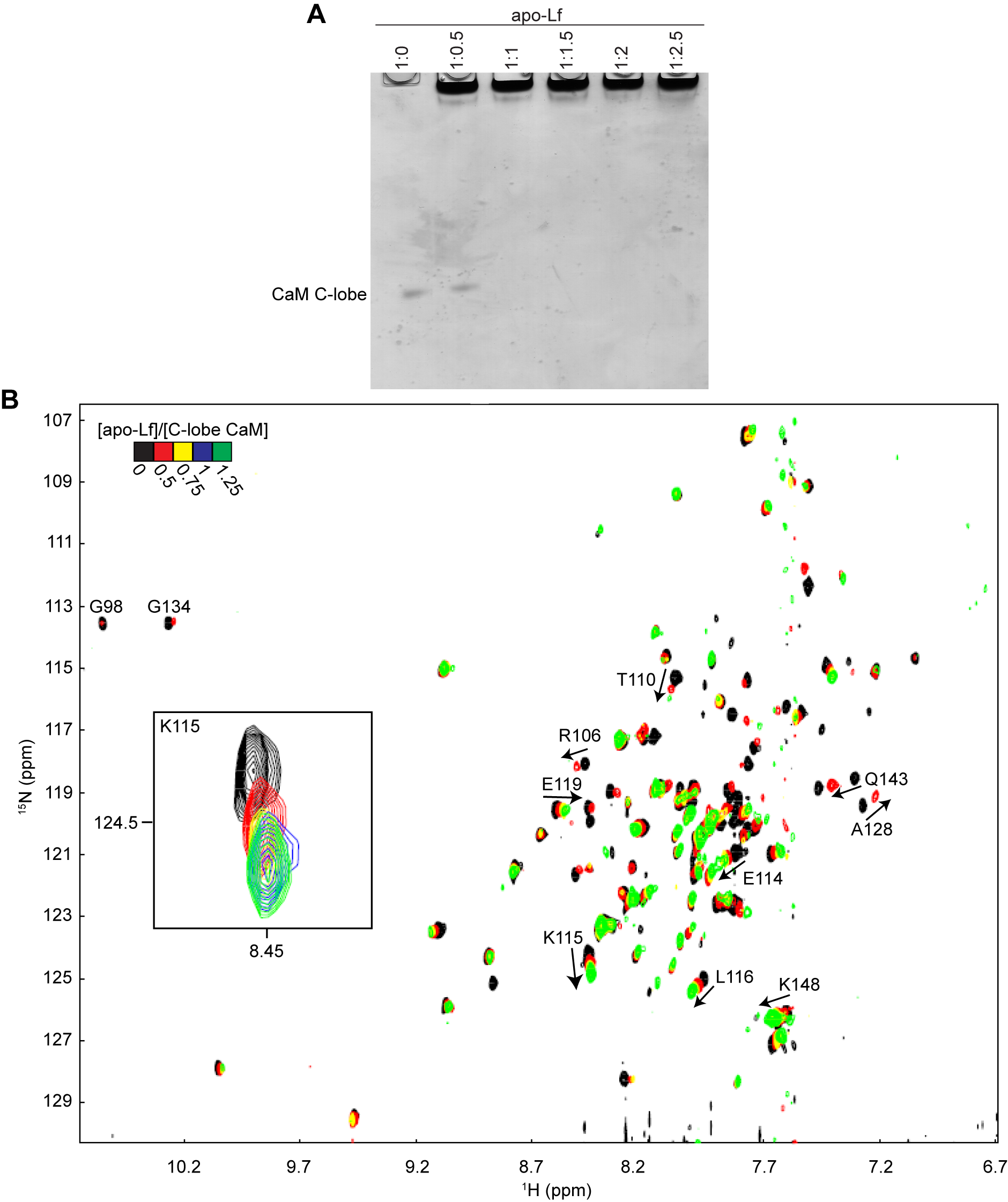

Supplement: Figure S2 — On its own, the C-terminal domain of CaM is sufficient to interact apo-Lf. A) Non-denaturing PAGE band shift analysis of the isolated C-terminal lobe Ca2+-CaM binding to apo-Lf. The ratio of CaM to apo-Lf is indicated above each lane. B) NMR titration data examining the binding of unlabeled apo-Lf to the isolated C-terminal lobe of 2H/15N-labeled Ca2+-CaM. TROSY-HSQC spectra were collected at titration steps corresponding to the [apo-Lf]/[CaM C-lobe] molar ratio of 0 (black), 0.5 (red), 0.75 (yellow), 1 (blue), 1.25 (green). These spectra are characteristic of an interaction in fast exchange, contrasting with the slow exchange observed in the titration of intact CaM. This difference in chemical exchange likely reflects the loss of the minor stabilizing contributions provided by the N-terminal lobe of CaM. (TIF) [file pone.0051026.s002.tif]
